# Impact of Two Common Xeroderma Pigmentosum Group D (XPD) Gene Polymorphisms on Risk of Prostate Cancer

**DOI:** 10.1371/journal.pone.0044756

**Published:** 2012-09-21

**Authors:** Yuanyuan Mi, Lifeng Zhang, Ninghan Feng, Sheng Wu, Xiaoming You, Hongbao Shao, Feng Dai, Tao Peng, Feng Qin, Jiangang Zou, Lijie Zhu

**Affiliations:** 1 Department of Urology, The Third Affiliated Hospital of Nantong University, Wuxi, Jiangsu, China; 2 Department of Urology, The Affiliated Changzhou No 2. Hospital of Nanjing Medical University, Changzhou, Jiangsu, China; 3 Department of Urology, The First Affiliated Hospital of Nanjing University, Nanjing, Jiangsu, China; The Chinese University of Hong Kong, Hong Kong

## Abstract

**Background:**

DNA repair genes (eg: xeroderma pigmentosum group D, XPD) may affect the capacity of encoded DNA repair enzymes to effectively remove DNA adducts or lesions, which may result in enhanced cancer risk. The association between XPD gene polymorphisms and the susceptibility of prostate cancer (PCa) was inconsistent in previous studies.

**Methodology/Principal Findings:**

A meta-analysis based on 9 independent case-control studies involving 3165 PCa patients and 3539 healthy controls for *XPD Gln751Lys* SNP (single nucleotide polymorphism) and 2555 cases and 3182 controls for *Asn312Asp* SNP was performed to address this association. Meanwhile, odds ratio (OR) and 95% confidence intervals (CIs) were used to evaluate this relationship. Statistical analysis was performed with STATA10.0. No significant association was found between *XPD Gln751Lys* SNP and PCa risk. On the other hand, in subgroup analysis based on ethnicity, associations were observed in Asian (eg. *Asn* vs. *Asp*: OR = 1.34, 95%CI = 1.16–1.55; *Asn/Asn+Asn/Asp* vs. *Asp/Asp*: OR = 1.23, 95%CI = 1.07–1.42) and African (eg. *Asn* vs. *Asp*: OR = 1.31, 95%CI = 1.01–1.70; *Asn/Asn* vs. *Asp/Asp*: OR = 1.71, 95%CI = 1.03–7.10) populations for *Asn312Asp* SNP. Moreover, similar associations were detected in hospital-based controls studies; the frequency of *Asn/Asn* genotype in early stage of PCa men was poorly higher than those in advanced stage of PCa men (OR = 1.45, 95%CI = 1.00–2.11).

**Conclusion/Significance:**

Our investigations demonstrate that *XPD Asn312Asp* SNP not the *Gln751Lys* SNP, might poorly increase PCa risk in Asians and Africans, moreover, this SNPs may associate with the tumor stage of PCa. Further studies based on larger sample size and gene-environment interactions should be conducted to determine the role of XPD gene polymorphisms in PCa risk.

## Introduction

Prostate cancer (PCa) is the most common male non-dermatological cancer in Europe and the USA, and the sixth leading cause of cancer related-deaths, accounting for 14% (903, 500) of total new diagnosed cancer cases and 6% (258, 400) of whole cancer deaths in males in 2008 [1]. Despite its high incidence and morbidity, the etiology of PCa remains largely unknown, only age, ethnicity, diet and a family history are established risk factors. It is well established that genetic factor also play an important role in pathogenesis of PCa [2], [3].

Various DNA alterations can be caused by exposure to environmental and endogenous carcinogens, including ultraviolet (UV) light, cigarette smoke, dietary factors, reactive oxygen species, and carcinogens. Most of these alterations, if not repaired, can result in genetic instability, mutagenesis and cell death. Because DNA repair pathways (DRP) play a critical role in maintaining the genomic integrity in general and specialized functions of cell as well as in the prevention of carcinogenesis, therefore the defection of those genes in DRP can lead to highter susceptibility to multiple cancers [4], [5].

There are a number of DRP, each responsible for repairing a different type of DNA damage. Base excision repair (BER) removes simple base modifications, including single-strand breaks, oxidative DNA damage, and alkylation and nonbulky adducts [6]. Nucleotide excision repair (NER) removes larger lesions, which often result from environmental damage, including UV radiation and external carcinogens [7]. Alkyltransferases directly reverse DNA damage by transferring alkyl groups from damaged DNA onto the transferase enzyme [8]. Double-stranded DNA breaks are repaired through mechanisms including the homologous recombination repair pathway [9]. Sequence variants in DNA repair genes also are thought to modulate DNA repair capacity and consequently may be associated with altered cancer risk [10].

The xeroderma pigmentosum group D (XPD) gene encoding for NER protein is located on chromosome 19q 13.3. It comprises 23 exons and spans about 54,000 base pairs [11], [12]. The XPD gene, also known as excision repair cross-complementing rodent repair deficiency Group 2 (ERCC2), is important in environmentally induced cancer [13]. XPD is an enzyme in the NER pathway that removes certain DNA cross-links, UV photo-lesions, and bulky chemical adducts [14]. Mutations in the XPD gene can completely prevent DNA opening and dual incision, steps that lead to the repair of DNA adducts [15].

Two common non-synonymous single-nucleotide polymorphisms (SNP) in the coding region of the XPD gene have been identified: a *G→A* substitution causing exon 10 codon *312 Asp* to be exchanged for *Asn* (*Asp312Asn*, *D312N*, *G23592A*, rs1799793) and an *A→C* substitution causing exon 23 codon *751 Lys* to be substituted for *Gln* (*Lys751Gln*, *L751Q*, *A35931C*, rs13181) [16], [17]. These two polymorphisms are associated with lower DNA repair capacity and a higher level of DNA adducts [17], [18].

Several epidemiologic studies have examined associations between SNPs in DNA repair genes, mostly non-synonymous SNPs in XPD gene with potential functional significance and risk of PCa [19]–[26]. However, results have been inconsistent across these studies due in part to different study populations, case ascertainment, or due to small sample sizes of each study and thus the potential for false-positive findings as well as limited power to detect modest associations. The objective of our study was to examine associations between two XPD polymorphisms and risk of PCa in larger samples by meta-analysis.

## Materials and Methods

### Search strategy

We searched the Pubmed and Embase databases for all articles on the association between XPD two polymorphisms (*Asn312Asp* and *Gln751Lys*) and PCa risk up to March 20, 2012. The medical subject headings and key words used for search were ‘XPD or ERCC2 or xeroderma pigmentosum group D or excision repair cross-complementing rodent repair deficiency Group 2’, and ‘prostate cancer or tumor’, and ‘polymorphism or variant’. The electronic searching was supplemented by checking reference lists from the identified articles and reviews for additional original reports. All of studies must meet the following criteria: (1) study was designed using the methodology of a case-control study; (2) the association between *XPD Asn312Asp* and/or *Gln751Lys* polymorphisms and PCa risk was explored; (3) cases with carcinomas were diagnosed by histopathology. The major exclusion criteria were: (1) duplicate data, (2) abstract, comment, review and editorial, and (3) no sufficient data were reported. All studies were published in English language.

### Data abstraction

Two of the authors (Dai, Peng) extracted all data independently, complied with the selection criteria, and reached a consensus on all items. In case of disagreement, a third author (Zhang) assessed the articles. The following items were collected: first author's last name, year of publication, country of origin, ethnicity, source of control (hospital-based, HB and population-based, PB) and Hardy–Weinberg equilibrium (HWE) of control, total number and genotype distributions in cases/controls and genotyping method. For studies including subjects of different ethnicities, data were extracted separately and categorized as Caucasians, Asians and Africans. Agalliu et al. [25] reported that PCa diagnosed with regional or distant stage were compared to men with localized stage at diagnosis for cancer stage. However, Mandal et al. [26] reported that tumor stage was divided into bone metastasis (+) and none bone metastasis (−). In our present study, we classified the tumor stage into ‘early stage’ [localized stage or none bone metastasis (−)] and ‘advanced stage’ [regional/distant stage or bone metastasis (+)]. With respect to the Gleason score analyses, cases were grouped into those with Gleason score of 2–7 (≤7), and those with Gleason score of 7–10 (≥7) at diagnosis.

### Statistical analysis

The strength of the association between the XPD two polymorphisms and PCa risk was measured by odds ratios (ORs) with 95% confidence intervals (CIs). Pooled ORs were obtained from combination of single studies by heterozygote comparison (*Gln/Gln* vs. *Gln/Lys* for *751* SNP, *Asn/Asn* vs. *Asn/Asp* for *312* SNP), homozygote comparison (*Gln/Gln* vs. *Lys/Lys* for *751* SNP, *Asn/Asn* vs. *Asp/Asp* for *312* SNP), dominant and recessive models (*Gln/Gln*+*Gln/Lys* vs. *Lys/Lys* and *Gln/Gln* vs. *Gln/Lys*+*Lys/Lys* for *751* SNP, *Asn/Asn*+*Asn/Asp* vs. *Asp/As*p and *Asn/Asn* vs. *Asn/Asp*+*Asp/Asp* for *312* SNP) and allelic comparison (*Gln vs.Lys* for *751* SNP, *Asn* vs. *Asp* for *312* SNP), respectively. The heterogeneity among the studies was checked by using the chisquare based *Q* statistic and considered statistically significant at *P*<0.05 [27]. When *P*>0.05, the pooled OR of each study was calculated by using the fixed-effects model (the Mantel-Haenszel method, which weights the studies by the inverse of the variance of estimates); otherwise, the random-effects model (the DerSimonian and Laird method) was used [28], [29]. The significance of the pooled OR was determined by the *Z*-test, and *P*<0.05 was considered statistically significant. The departure of frequencies of XPD polymorphisms from expectation under HWE was assessed by the chi-square test in controls and a *P*<0.05 was considered as significant disequilibrium. Publication bias was diagnosed with Egger's linear regression method and funnel plot. The *P*-value less than 0.05 in Egger's linear regression indicated the presence of potential publication bias [30], [31]. All statistical tests for this meta-analysis were performed with STATA software, version 10.0 (STATA Corp., College Station, TX, USA), and all tests were two-sided.

## Results

### Studies characteristics

**Figure 1:**
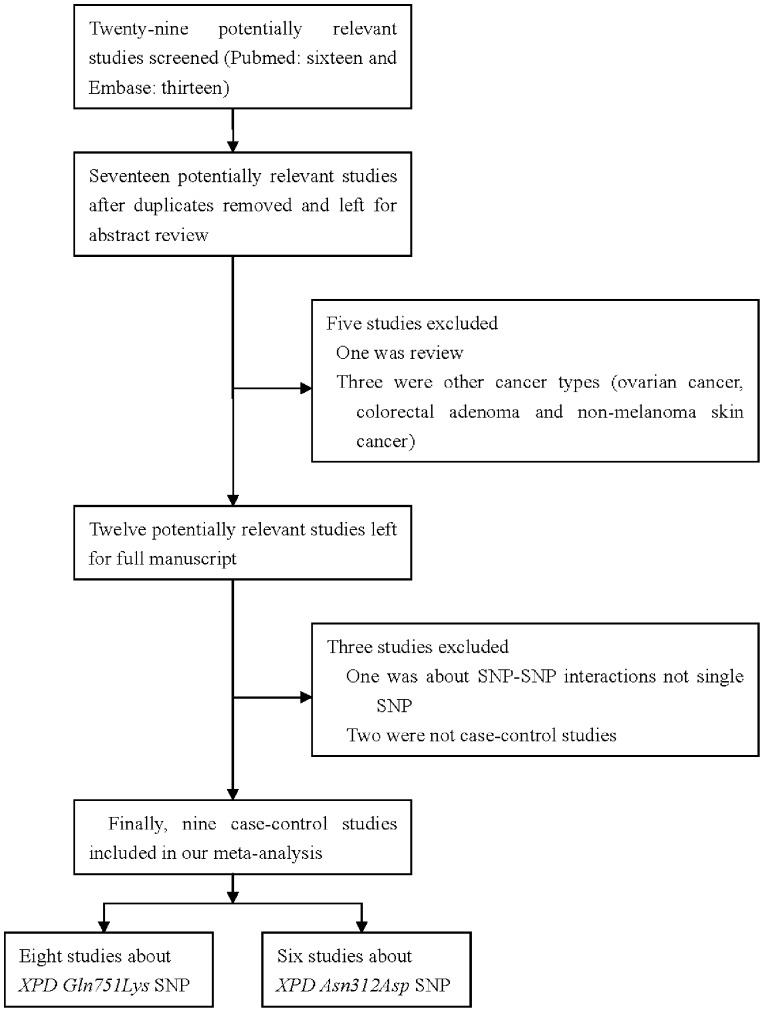
Flow chart of selection studies in our meta-analysis.

A total of 29 articles were achieved by literature search from the Pubmed and Embase, using different combinations of key terms. As shown in **Figure 1**, nine eligible studies were retrieved for detailed evaluation. We excluded 20 studies: 12 were reduplicate, one article type was review, 3 papers were about other cancer types (ovarian cancer, colorectal adenoma and non-melanoma skin cancer), two were not relate to case-control studies and last article was about SNP-SNP interactions not for single SNP polymorphism. Finally, we identified 8 articles (9 case-control studies, 3165 cases and 3539 controls) for *Gln751Lys* polymorphism and 6 articles (7 case-control studies, 2555 cases and 3182 controls) for *Asn312Asp* polymorphism to evaluate the association with risk for PCa, respectively (**Table S1**). The distribution of genotypes among controls was consistent with HWE in all studies except two [16], [19]. To our regret, in all of included studies, the diet and a family history of PCa were not established. With the exception of two studies (Gao et al and Lavender et al), the age between cases and controls was matched. Control population including all consisted of study participants with a normal digital rectal examination (DRE) and serum prostatic specific antigen (PSA) values of <4 ng/ml, as well as old matched men, not cancer family history and not previous cancer history. Rybicki et al. [16] reported a case-control study that 90% were Caucasians, 9% were African-Americans, and only 1% were Asians or Hispanics, we selected the primarily Caucasians in our meta. For the Gln751Lys polymorphism, three studies had been conducted on Caucasians, four on Asians, two studies on Africans. Two studies were HB; the others were PB. For the Asn312Asp polymorphism, there had 3 studies about Caucasians, 2 about Africans and 2 about Asians; three studied came from HB and 4 from PB. Two studies [25], [26] refered to Gleason scoring system and clinical tumor stage (**Table 1**). The genotyping methods included PCR-FLIP (polymerase chain reaction and restrictive fragment length polymorphism), ABI SNPlex (Applied Biosystems SNPlex™ Genotyping system), ARM-PCR (amplification refractory mutation-specific polymerase chain reaction) and MALDI-TOF-MS (matrix-assisted laser desorption ionization time of flight mass spectrometry).

**Table 1:** The number of different genotypes of two polymorphisms of XPD gene in PCa cases and the relationship in clinical characteristics.

XPD	First author	Cases(M/M+M/W+W/W)			
		Gleason≥7	Gleason≤7	Early stage	Advanced stage
**Gln751Lys**	Agalliu	1036(126+492+418)	193(27+82+84)	964(125+450+389)	269(28+125+116)
	Mandal	102(7+45+50)	121(7+61+53)	79(7+36+36)	72(5+37+30)
**Asn312Asp**	Agalliu	1043(103+490+450)	193(17+84+92)	970(100+448+422)	270(20+127+123)
	Mandal	102(24+31+47)	121(25+44+52)	79(21+24+34)	72(12+26+34)
Positive results					
	Genotype	Early stage	Advanced stage	OR(95%CI)/*Q*-test	*Z*-test
**Asn312Asp**	Asn/Asn	121	32		
	Asn/Asp+Asp/Asp	928	310	1.45(1.00–2.11)/0.732	z = 1.95,p = 0.052

### Quantitative synthesis

The results of the overall meta-analysis did not suggest any associations between two XPD (*Asn312Asp/Gln751Lys*) polymorphisms and PCa susceptibility for all genetic models (eg: homozygote comparison: OR = 0.99/1.48, 95%CI = 0.86–1.14/0,90–2.43, *P*_heterpgeneity_ = 0.681/0.000, *P* = 0.926/0.118; dominant model: OR = 1.00/1.04, 95%CI = 0.95–1.04/0,99–1.09, *P*_heterpgeneity_ = 0.955/0.064, *P* = 0.887/0.159; allelic comparison: OR = 1.00/1.20, 95%CI = 0.95–1.05/0,99–1.46, *P*_heterpgeneity_ = 0.769/0.001, *P* = 0.899/0.068, respectively)(**Table S2**). After excluding two studies not agreement with HWE, the overall association was not changed. We did not find any significant results when we stratified the studies by ethnicity and source of the controls between *Gln751Lys* polymorphism and PCa risk.

For *Asn312Asp* polymorphism, when stratified according to ethnicity, significantly increased associations were found in Asians (eg: *Asn vs. Asp*: OR = 1.34, 95%CI = 1.16–1.55, *P*_heterpgeneity_ = 0.619, *P* = 0.000; *Asn/Asn* vs. *Asp/Asp*: OR = 1.77, 95%CI = 1.29–2.42, *P*_heterpgeneity_ = 0.415, *P* = 0.000 and *Asn/Asn*+*Asn/Asp* vs. *Asp/Asp*: OR = 1.23, 95%CI = 1.07–1.42, *P*_heterpgeneity_ = 0.096, *P* = 0.005) and Africans (*Asn* vs. *Asp*: OR = 1.31, 95%CI = 1.01–1.70, *P*_heterpgeneity_ = 0.885, *P* = 0.046; *Asn/Asn* vs. *Asp/Asp*: OR = 1.71, 95%CI = 1.03–7.10, *P*_heterpgeneity_ = 0.617, *P* = 0.043 and *Asn/Asn* vs. *Asn/Asp*+*Asp/Asp*: OR = 2.63, 95%CI = 1.00–6.89, *P*_heterpgeneity_ = 0.609, *P* = 0.050). Similar relationships were reported in HB source of the controls subgroup (**Table S2**).

Interestingly, compared to *Asn/Asp*+*Asp/Asp* genotypes, PCa cases with *Asn/Asn* genotype showed poorly higher percentage value in early stage group, but not in advanced stage group (OR = 1.45, 95%CI = 1.00–2.11, *P*_heterpgeneity_ = 0.732, *P* = 0.052)(**Table 1**). To our regret, no relationship was found between *Gln751Lys* SNP and Gleason score or tumor stage of PCa, also between *Asn312Asp* SNP and Gleason score of PCa (data not shown).

### Sensitivity analysis and bias diagnosis

We use sensitivity analysis to determine whether modification of the inclusion criteria of the meta-analysis affected the final results. Finally, no other single study influenced the summary OR qualitatively as indicated by sensitivity analysis (data not show). The Egger's test was performed to access the publication bias of literatures, which was used to provide statistical evidence of funnel plot symmetry. Ultimately, the results did not suggest any evidence of publication bias (**Table S3**).

## Discussion

The identification of SNPs that affect gene function or expression and contribute to PCa susceptibility is important to help predict individual and population risk and understand the pathogenesis of PCa. Rybicki et al. first found XPD two common polymorphisms to be associated with the risk of PCa in a Caucasian population in 2004 [16]. After that, several investigators duplicated his work in different populations. However, the results remained confusing, even within the same population. Meta-analysis is a means of increasing the effective sample size under investigation through the pooling of data from individual association studies, thus enhancing the statistical power of the analysis for the estimation of genetic effects [32]. The two XPD polymorphisms have been associated with the risk of head and neck cancer [33], esophageal cancer [34], lung cancer [35] and breast cancer [36]. However, the relationship between these two polymorphisms and PCa susceptibility is undetermined. In our present study, we analyzed the associations between XPD two SNPs (*Asn312Asp/Gln751Lys*) and PCa risk by using meta method to get a powerful conclusion. The potential role of XPD two polymorphisms as determinant of PCa risk was investigated in a sample of 6752 subjects (3165 cases and 3539 controls for *Gln751Lys* and 2555 cases and 3182 controls for *Asn312Asp*) from nine published case-control studies.

Little is know for sure about the underlying mechanism for this association. The XPD gene has been mapped in chromosome 19q13.3. It spans over 20 kb, contains 23 exons and encodes the 761-amino acid protein. The XPD protein, on one hand, possesses both single-strand DNA-dependant ATPase and 5′-3′ DNA helicase activities, which is essential for NER pathway and transcription; on the other hand, is absolutely necessary for efficient DNA repair capacity, which is essential for the maintenance of genetic stability and associated with cancer susceptibility when incompetent [13], [37]. Two common SNPs in XPD gene are in linkage disequilibrium, and their mutant phenotypes have shown to be associated with lower DNA repair capacity [18], which means these SNPs may contribute to carcinogenesis and can be as risk factors for cancer development. Many epidemiological studies have investigated the association between two XPD polymorphisms and PCa, but the results were inconclusive. Bau et al. [19] reported a 1.81-fold increased PCa risk in individuals who carry at least 1 mutant allele for the *Asn312Asp* or *Gln751Lys* polymorphism. Similarly, Rybicki et al. [16] also reported a modest increase in PCa risk (OR 1.16 and 1.14) in individuals if they are carriers of the rarer variant genotype (*312Asn/Asn* or *751Gln/Gln*, respectively). Lavender et al. [24] found that individuals possessing at least one XPD 312 Asn allele had a 1.3- to 8.6-fold higher PCa risk when compared to those with the *312 Asp/Asp* genotype, however, there were no significant differences in the allele frequencies between cases and controls for *Gln751Lys* polymorphism. Our study showed similar observation with Lavender et al. [24] and Mandal et al. [26].

In present study, no significant association was found under any genetic model in the overall analysis. However, in the stratified analysis by ethnicity, significant increased associations were detected between *XPD Asn312Asp* not *Gln751Lys* polymorphism and PCa among Asian and African populations. There must be some factors that would contribute to this discrepancy. First, multiple genes and environmental factors may lead to PCa formation. Second, ethnicity may be related to PCa incidence. For example, PCa is the most common non-cutaneous malignancy and the second leading cause of cancer mortality in Western men. In contrast to the trends of western countries, incidence and mortality rates are rising in several Asian and central/eastern-European countries, such as Japan, China and Poland, suggesting an increasingly westernized lifestyle in these regions [38], [39]. Finally, time lag bias and publication bias may also play a role in this meta-analysis.

We all know that the Gleason score grading system has withstood the test of time as a predictor of PCa outcome. Additionally, the tumor stage of PCa is central to management because it contributes both to predicting prognosis and planning treatment. In the current study, the frequency of PCa cases carried *312Asn/Asn* genotype in early stage cases was more higher than in advanced stage cases, in addition, *Asn/Asn* genotype was associated with increased PCa risk, thus, we predicted *312Asn/Asn* genotype could poorly help us to detect PCa and evaluate its prognosis. No association was detected between clinical characteristics of PCa and *Gln751Lys* SNP.

Although, we have put considerable efforts and resources into testing possible association between XPD polymorphisms and PCa risk, there are still some limitations inherited from the published studies. First, although we collected all the eligible studies, the sample size of the included studies was not large enough, which could increase the likehood of type I and type II errors. Therefore, there was a lack of statistical power to better evaluate the association between XPD polymorphisms and PCa risk, especially in subgroup analysis by ethnicity. Second, gene-gene and gene-environment interactions were not analyzed. It is possible that specific environmental and lifestyle factors may alter those associations between gene polymorphisms and PCa. Therefore, it is necessary to evaluate the roles of some special environment factors and lifestyles such as diet, alcohol consumption and smoking status and cancer family history. Third, two studies refered to Gleason score for *Gln751Lys* and also two studies about tumor stage about *Asn312Asp*, the number of which was small, further studies should increase these relations to enlarge the subsequent meta-analysis. Fourth, we classified the tumor stage into ‘early stage’ [localized stage or none bone metastasis (−)] and ‘advanced stage’ [regional/distant stage or bone metastasis (+)], however, the criteria of patient's selection in these two studies were not quite concordant. In spite of these, our meta-analysis also had three advantages. First, substantial number of cases and controls were pooled from different studies, which significantly increased statistical power of the analysis. Second, the quality of case–control studies included in the current meta-analysis was satisfactory based on our selection criteria. Third, the control groups were all healthy people.

In conclusion, present analysis suggested that *XPD Asn312Asp* not *Gln751Lys* polymorphism may contribute to genetic susceptibility for increased PCa risk in Africans and Asians. Furthermore, *XPD Asn312Asp* polymorphism was associated with PCa prognosis and/or outcome. Large population studies are needed to be performed to clarify possible roles of XPD polymorphisms in the etiology and clinical characteristics of PCa.

## Supporting Information

Table S1 Study characteristics from published studies on the relationship between XPD gene two polymorphisms and PCa.
(DOC)

Table S2 Total and stratified analysis of XPD gene two polymorphisms on PCa.
(DOC)

Table S3 Publication bias tests (Egger's funnel plot for publication bias test) for XPD gene two polymorphisms.
(DOC)
