# Supplementary material for: Impact of Two Common Xeroderma Pigmentosum Group D (XPD) Gene Polymorphisms on Risk of Prostate Cancer
Source: PLoS One. 2012 Sep 21;7(9):e44756. doi: 10.1371/journal.pone.0044756 (PMC3448601; doi:10.1371/journal.pone.0044756)
Supplement: Table S1 — Study characteristics from published studies on the relationship between XPD gene two polymorphisms and PCa. (DOC) [file pone.0044756.s001.doc]

**Table S1 Study characteristics from published studies on the relationship between XPD gene two polymorphisms and PCa**.

| First author | Year | Country | Ethnicity | Source of | Cases |  | Controls | Genotyping method |
| --- | --- | --- | --- | --- | --- | --- | --- | --- |
|  |  |  |  | Control/HWE | Total(M/M+M/W+W/W) |  | Total(M/M+M/W+W/W) |  |
| **Gln751Lys** |  |  |  |  |  |  |  |  |
| Sobti | 2012 | India | Asian | PB/0.530 | 150(21+67+62) |  | 150(14+69+67) | PCR-FLIP |
| Gao | 2010 | USA | Caucasian | PB/0.613 | 428(64+178+186) |  | 118(13+56+49) | Nucleotide Sequencing |
| Lavender | 2010 | USA | African | HB/0.076 | 183(13+60+110) |  | 599(38+194+367) | TaqMan |
| Agalliu | 2010 | USA | Caucasian | PB/0.733 | 1233(153+575+505) |  | 1228(177+571+480) | ABI SNPlex |
| Agalliu | 2010 | USA | African | PB/0.686 | 146(11+48+87) |  | 83(5+28+50) | ABI SNPlex |
| Mandal | 2010 | India | Asian | PB/0.258 | 171(14+84+73) |  | 200(17+94+89) | ARM-PCR |
| Bau | 2007 | China-Taiwan | Asian | HB/0.000 | 123(2+10+111) |  | 479(5+33+441) | PCR-FLIP |
| Ritchey | 2005 | USA | Asian | PB/0.817 | 160(0+19+141) |  | 247(1+33+213) | MALDI-TOF-MS |
| Rybicki | 2004 | USA | Caucasian | PB/0.547 | 571(75+273+223) |  | 435(52+205+178) | PCR-FLIP |
| **Asn312Asp** |  |  |  |  |  |  |  |  |
| Dhillon | 2011 | Australia | Caucasian | HB/0.188 | 116(8+37+71) |  | 132(10+42+80) | PCR-FLIP |
| Lavender | 2010 | USA | African | HB/0.568 | 190(5+39+146) |  | 631(5+116+510) | TaqMan |
| Agalliu | 2010 | USA | Caucasian | PB/0.068 | 1240(120+575+545) |  | 1221(166+528+527) | ABI SNPlex |
| Agalliu | 2010 | USA | African | PB/0.332 | 144(7+31+106) |  | 82(2+15+65) | ABI SNPlex |
| Mandal | 2010 | India | Asian | PB/0.569 | 171(39+56+76) |  | 200(20+81+99) | ARM-PCR |
| Bau | 2007 | China-Taiwan | Asian | HB/0.000 | 123(22+39+62) |  | 479(63+106+310) | PCR-FLIP |
| Rybicki | 2004 | USA | Caucasian | PB/0.018 | 571(72+269+230) |  | 437(39+218+180) | PCR-FLIP |

HB: hospital-based; PB: population-based; M: Gln for 751 SNP, Asn for 312 SNP; W: Lys for 751 SNP, Asp for 312 SNP; PCR-FLIP: polymerase chain reaction and restrictive fragment length polymorphism; ABI SNPlex: Applied Biosystems SNPlex™ Genotyping system; ARM-PCR: amplification refractory mutation-specific polymerase chain reaction; MALDI-TOF-MS: matrix-assisted laser desorption ionization time of flight mass spectrometry
