# Supplementary material for: Impact of Two Common Xeroderma Pigmentosum Group D (XPD) Gene Polymorphisms on Risk of Prostate Cancer
Source: PLoS One. 2012 Sep 21;7(9):e44756. doi: 10.1371/journal.pone.0044756 (PMC3448601; doi:10.1371/journal.pone.0044756)
Supplement: Table S2 — Total and stratified analysis of XPD gene two polymorphisms on PCa. (DOC) [file pone.0044756.s002.doc]

**Table S2 Total and stratified analysis of XPD gene two polymorphisms on PCa**.

| Variables | N | Cases/ | Allelic contrast |  | Homozygote comparison |  | Heterozygote comparison |  | Dominant genetic model |  | Recessive genetic model |
| --- | --- | --- | --- | --- | --- | --- | --- | --- | --- | --- | --- |
|  |  | Controls | OR(95%CI) *P*^b^/*P*^c^ |  | OR(95%CI) *P*^b^/*P*^c^ |  | OR(95%CI) *P*^b^/*P*^c^ |  | OR(95%CI) *P*^b^/*P*^c^ |  | OR(95%CI) *P*^b^/*P*^c^ |
| **Gln751Lys** |  |  |  |  |  |  |  |  |  |  |  |
| Total | 9 | 3165/3539 | 1.00(0.95-1.05)0.769/0.899 |  | 0.99(0.86-1.14)0.681/0.926 |  | 1.00(0.94-1.05)0.987/0.862 |  | 1.00(0.95-1.04)0.955/0.887 |  | 1.00(0.86-1.16)0.658/0.972 |
| HWE | 8 | 3042/3060 | 0.99(0.94-1.05)0.752/0.823 |  | 0.99(0.86-1.14)0.615/0.883 |  | 0.99(0.94-1.05)0.982/0.804 |  | 0.99(0.95-1.04)0.949/0.809 |  | 0.99(0.86-1.15)0.586/0.933 |
| Ethnicity |  |  |  |  |  |  |  |  |  |  |  |
| Asian | 4 | 604/1076 | 1.06(0.92-1.22)0.691/0.441 |  | 1.22(0.80-1.86)0.779/0.353 |  | 1.02(0.88-1.19)0.919/0.749 |  | 1.04(0.91-1.18)0.845/0.953 |  | 1.21(0.78-1.88)0.743/0.389 |
| African | 2 | 329/682 | 1.04(0.87-1.25)0.983/0.642 |  | 1.16(0.69-1.93)0.878/0.582 |  | 1.01(0.83-1.23)0.894/0.905 |  | 1.03(0.86-1.22)0.947/0.771 |  | 1.15(0.68-1.95)0.855/0.590 |
| Caucasian | 3 | 2232/1781 | 0.98(0.92-1.04)0.355/0.492 |  | 0.95(0.81-1.11)0.236/0.506 |  | 0.99(0.93-1.05)0.623/0.669 |  | 0.98(0.93-1.04)0.586/0.567 |  | 0.95(0.81-1.13)0.200/0.582 |
| Source of control |  |  |  |  |  |  |  |  |  |  |  |
| HB | 2 | 306/1078 | 1.07(0.88-1.31)0.538/0.507 |  | 1.17(0.67-2.05)0.703/0.576 |  | 1.04(0.83-1.30)0.678/0.715 |  | 1.05(0.87-1.28)0.591/0.592 |  | 1.16(0.66-1.06)0.710/0.599 |
| PB | 7 | 2859/2461 | 0.99(0.94-1.05)0.674/0.727 |  | 0.98(0.85-1.13)0.523/0.797 |  | 0.99(0.94-1.05)0.963/0.750 |  | 0.99(0.94-1.04)0.914/0.726 |  | 0.99(0.85-1.15)0.488/0.856 |
| **Asn312Asp** |  |  |  |  |  |  |  |  |  |  |  |
| Total | 7 | 2555/3182 | 1.20(0.99-1.46)0.001/0.068 |  | 1.48(0.90-2.43)0.000/0.118 |  | 1.04(0.98-1.11)0.216/0.238 |  | 1.04(0.99-1.09)0.064/0.159 |  | 1.44(0.88-2.36)0.000/0.151 |
| HWE | 5 | 1861/2266 | 1.16(0.90-1.50)0.009/0.258 |  | 1.50(0.70-3.23)0.000/0.300 |  | 1.03(0.95-1.11)0.882/0.453 |  | 1.02(0.96-1.08)0.487/0.582 |  | 1.50(0.68-3.33)0.000/0.319 |
| Ethnicity |  |  |  |  |  |  |  |  |  |  |  |
| Asian | 2 | 294/679 | 1.34(1.16-1.55)0.619/0.000 |  | 1.77(1.29-2.42)0.415/0.000 |  | 1.29(0.64-2.59)0.030/0.481 |  | 1.23(1.07-1.42)0.096/0.005 |  | 1.74(1.25-2.43)0.128/0.001 |
| African | 2 | 334/713 | 1.31(1.01-1.70)0.885/0.046 |  | 1.71(1.03-7.10)0.617/0.043 |  | 1.16(0.87-1.53)0.856/0.309 |  | 1.23(0.94-1.59)0.861/0.126 |  | 2.63(1.00-6.89)0.609/0.050 |
| Caucasian | 3 | 1927/1790 | 0.97(0.91-1.04)0.183/0.365 |  | 0.96(0.55-1.67)0.020/0.889 |  | 1.01(0.95-1.08)0.854/0.726 |  | 0.99(0.94-1.05)0.897/0.843 |  | 0.96(0.53-1.74)0.007/0.899 |
| Source of control |  |  |  |  |  |  |  |  |  |  |  |
| HB | 3 | 429/1242 | 1.25(1.07-1.46)0.180/0.004 |  | 1.50(1.04-2.15)0.225/0.028 |  | 1.12(1.01-1.46)0.174/0.043 |  | 1.22(1.05-1.43)0.177/0.011 |  | 1.37(0.94-1.99)0.247/0.100 |
| PB | 4 | 2126/1940 | 1.14(0.90-1.44)0.007/0.292 |  | 1.39(0.70-2.75)0.000/0.341 |  | 1.01(0.95-1.08)0.803/0.730 |  | 1.01(0.95-1.07)0.566/0.740 |  | 1.41(0.69-2.88)0.000/0.346 |

^a^ Number of comparisons, ^b^ *P* value of Q-test for heterogeneity test, ^c^ *P*-value of Z-test for significant test
