# Supplementary material for: Impact of Two Common Xeroderma Pigmentosum Group D (XPD) Gene Polymorphisms on Risk of Prostate Cancer
Source: PLoS One. 2012 Sep 21;7(9):e44756. doi: 10.1371/journal.pone.0044756 (PMC3448601; doi:10.1371/journal.pone.0044756)
Supplement: Table S3 — Publication bias tests (Egger's funnel plot for publication bias test) for XPD gene two polymorphisms. (DOC) [file pone.0044756.s003.doc]

**Table S3 Publication bias tests (Egger’s funnel plot for publication bias test) for XPD gene two polymorphisms**

| Genetic type | Coefficient | Standard error | t | *P* value | 95%CI of intercept |
| --- | --- | --- | --- | --- | --- |
| **Gln751Lys** |  |  |  |  |  |
| Allelic contrast | 0.747 | 0.417 | 1.79 | 0.116 | (-0.239,1.733) |
| Homozygote comparison | 0.763 | 0.408 | 1.87 | 0.104 | (-0.202,1.727) |
| Heterozygote comparison | 0.132 | 0.301 | 0.44 | 0.674 | (-0.579,0.843) |
| Dominant genetic model | 0.406 | 0.324 | 1.25 | 0.25 | (-0.360,1.173) |
| Recessive genetic model | 0.779 | 0.426 | 1.83 | 0.11 | (-0.228,1.787) |
| **Asn312Asp** |  |  |  |  |  |
| Allelic contrast | 2.773 | 1.099 | 2.52 | 0.053 | (-0.052,5.599) |
| Homozygote comparison | 2.677 | 1.219 | 2.2 | 0.079 | (-0.457,5.811) |
| Heterozygote comparison | 0.844 | 0.775 | 1.09 | 0.326 | (-1.149,2.837) |
| Dominant genetic model | 1.599 | 0.751 | 2.13 | 0.086 | (-0.330,3.530) |
| Recessive genetic model | 2.674 | 1.295 | 2.07 | 0.094 | (-0.654,6.003) |
